# Network Pharmacology-Based Analysis of the Effects of *Corydalis decumbens* (Thunb.) Pers. in Non-Small Cell Lung Cancer

**DOI:** 10.1155/2021/4341517

**Published:** 2021-08-21

**Authors:** Jun Liu, Zhibo He, Shan Li, Wenan Huang, Zhongjie Ren

**Affiliations:** ^1^Medical School, Jiujiang University, Jiujiang 332005, China; ^2^School of Literature and Communication, Jiujiang University, Jiujiang 332005, China

## Abstract

Non-small cell lung cancer (NSCLC) is one of the most malignant tumors worldwide. The main treatment for NSCLC is based on Western medicine; however, the overall effect is unsatisfactory. This study aimed to investigate the potential therapeutic targets and pharmacological mechanisms of action of the traditional Chinese medicine *Corydalis decumbens* (Thunb.) Pers. in NSCLC based on network pharmacology and bioinformatics. The overlapping genes between *Corydalis decumbens* (Thunb.) Pers. and NSCLCs were screened using Venn analysis. Cytoscape 3.7.1 software was used to analyze the overlapping target protein-protein interaction (PPI) network. Gene ontology and pathway enrichment analysis using the Kyoto Encyclopedia of Genes and Genomics database were performed to exploring biological functions of the overlapping genes. The gene expression profiling interactive analysis dataset was used to analyze the correlation between hub gene expression and disease. This study revealed 38 nodes with 191 edges, which may be therapeutic targets for NSCLC. PPI network analysis showed that the most likely association was between the genes *AR* and *NCOA2*, *NCOA2*, and *RXRA* and *ESR1* and *NCOA2*. These overlapping genes were mainly enriched in the estrogen signaling pathway, calcium signaling pathway, cholinergic synapse, and PI3K-Akt signaling pathway. *ESR2* mRNA levels were signiﬁcantly downregulated in patients with lung adenocarcinoma (LUAD) getting worse, and *KDR* levels were lower in lung squamous cell carcinoma (LUSC) than those in normal tissue. *PTGS2* expression was correlated with the median survival time of LUAD, and *ESR1* expression was correlated with the median survival time of LUSC. The application of network pharmacology revealed the potential mechanism underlying the effects of *Corydalis decumbens* (Thunb.) Pers. in NSCLC treatment and provided a theoretical basis for further in-depth research in this field.

## 1. Introduction

Lung cancer is one of the most malignant tumors with high morbidity and mortality rates worldwide. It is divided into non-small cell lung cancer (NSCLC) and small cell lung cancer (SCLC) based on its different morphological characteristics. NSCLC accounts for 80% of all lung cancer cases and mainly includes lung adenocarcinoma (LUAD), lung squamous cell carcinoma (LUSC), and large cell lung cancer [1]. Recent research showed that the main treatment for NSCLC is based on Western medicine, including surgery, chemotherapy, radiotherapy, targeted therapy, and immunotherapy [2, 3]. However, the treatment for this disease is often accompanied by unsatisfactory outcomes, such as adverse reactions, easy recurrence and metastasis, poor prognosis, and high costs [4]. Thus, actively exploring safe and effective treatment methods is necessary to prevent and treat lung cancer [5].

Traditional Chinese medicine (TCM) has a history of more than 2000 years in China and mainly includes botanical, animal, and mineral medicines. It has been widely used to prevent and treat diseases, particularly in Asia [6, 7]. Studies have shown that Chinese herbal medicines can directly inhibit malignant tumor growth and proliferation [6]. For example, botanical ingredients such as alkaloids, dietary polyphenols, and saponins are the main inhibitors of lung cancer [8–10]. *Corydalis decumbens* (Thunb.) Pers. is botanical medicine that is rich in alkaloids and has mainly focused on treating cardiovascular and cerebrovascular diseases, arthritis, and myopia [11–13]; however, its use has been scarce in tumors. The current study, using network pharmacology and bioinformatics, aimed to address the following questions: does *Corydalis decumbens* (Thunb.) Pers. play role in treating NSCLC? Which ingredients of *Corydalis decumbens* (Thunb.) Pers. have an anti-NSCLC effect? What is the molecular mechanism underlying the effect of *Corydalis decumbens* (Thunb.) Pers. in NSCLC treatment?

## 2. Materials and Methods

### 2.1. Ingredients of *Corydalis decumbens* (Thunb.) Pers

We searched the active ingredients of *Corydalis decumbens* (Thunb.) Pers. using the Traditional Chinese Medicine Systems Pharmacology Database and Analysis Platform (TCMSP, http://tcmspw.com/tcmsp.php). The Chinese pinyin name “xiatianwu” was entered in the “herb name” column. The filter conditions were oral bioavailability (OB) ≥ 30% and drug similarity (DL) ≥ 0.18.

### 2.2. Drug Ingredient Target Network Construction

The corresponding targets of the active ingredients were obtained from TCMSP, SymMap (https://www.symmap.org/), and the Encyclopedia of Traditional Chinese Medicine (ETCM, http://www.tcmip.cn/ETCM/index.php/Home/Index/index.html). The reviewed human database from UniPort (http://www.uniport.org/) was used to translate target names to gene names. Cytoscape 3.7.1 software was used to construct the drug ingredients target network.

### 2.3. Potential Target Genes of *Corydalis decumbens* (Thunb.) Pers. against Lung Cancer

We used online tools such as GeneCards (https://www.Genecards.org) to identify the symbols of genes reported in NSCLC using keyword “NSCLC.” UniProt online databases were used to translate symbol names to gene names. The overlapping genes of *Corydalis decumbens* (Thunb.) Pers. and NSCLC were identified by drawing a Venn diagram. The overlapping genes were considered as potential *Corydalis decumbens* (Thunb.) Pers. genes playing a role in treating NSCLC. Cytoscape 3.7.1 software was used to construct the ingredients-potential target network.

### 2.4. Construction of the Overlapping Target Protein-Protein Interaction (PPI) Network

The STRING database (http://www.string-db.org/) and Cytoscape 3.7.1 software were used to elucidate the molecular mechanism underlying the anti-NSCLC effect of *Corydalis decumbens* (Thunb.) Pers. According to the STRING database with the species limited to “*Homo sapiens*” and a confidence score >0.4, the interaction proteins were screened. The PPI network was constructed using Cytoscape 3.7.1 for further visualization and analysis (PPI node degree, node interaction scores, and hub genes).

### 2.5. Functional Enrichment Analysis

The Database for Annotation, Visualization and Integrated Discovery (DAVID, https://david.ncifcrf.gov) was used for functional enrichment analysis of potential genes. “*Homo sapiens*” was selected as the species and “OFFICIAL GENE SYMBOL” was used for identification. Gene ontology (GO) functions and the Kyoto Encyclopedia of Genes and Genomics (KEGG) pathway enrichment analysis in the DAVID online tool were performed for exploring the biological function of the genes used in the treatment of NSCLC; *P* < 0.05 was considered as the cutoff criterion. In particular, GO functions include biological process (BP), molecular function (MF), and cellular component (CC). *P* < 0.01 was used as the cutoff criterion.

### 2.6. Correlation Analysis of Hub Gene Expression and Disease

The top 10 hub genes in the PPI network were verified using the Cytoscape 3.7.1 plugin cytoHubba. The gene expression profiling interactive analysis dataset (GEPIA, http://gepia.cancer-pku.cn/) was used to analyze the expression of hub genes, such as the differential expression analysis between NSCLC tissues and normal tissues, differential expression analysis according to pathological stages of cancer, and the expression analysis associated with overall survival (OS) of the NSCLC patients (*P* < 0.05). “MCC” was used as the calculate mode, and the “display the shortest path” as the displayed options, LUAD and LUSC, were selected as the cancer type dataset. Log2 FC cutoff = 2, *q* value cutoff = 0.05, and the other options were set as the default values.

## 3. Results

### 3.1. Potential Target Genes of *Corydalis decumbens* (Thunb.) Pers

**Figure 1:**
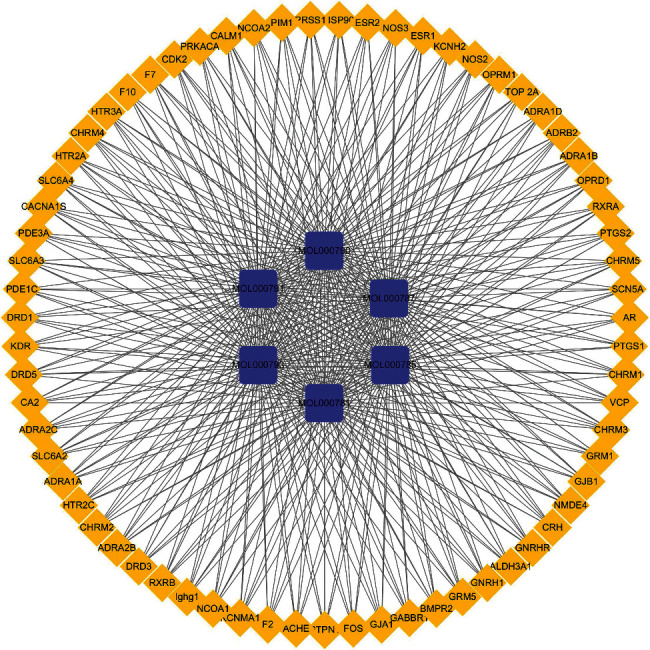
The ingredient-target network in the current study. The yellow diamond represents targets, and the purple rectangle represents the ingredients of *Corydalis decumbens* (Thunb.) Pers. The line between the two nodes represents the interaction.

**Figure 2:**
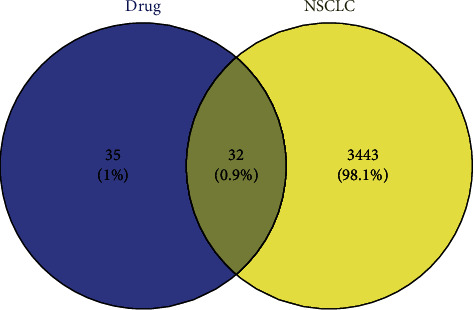
The overlapping genes in the Venn diagram.

**Figure 3:**
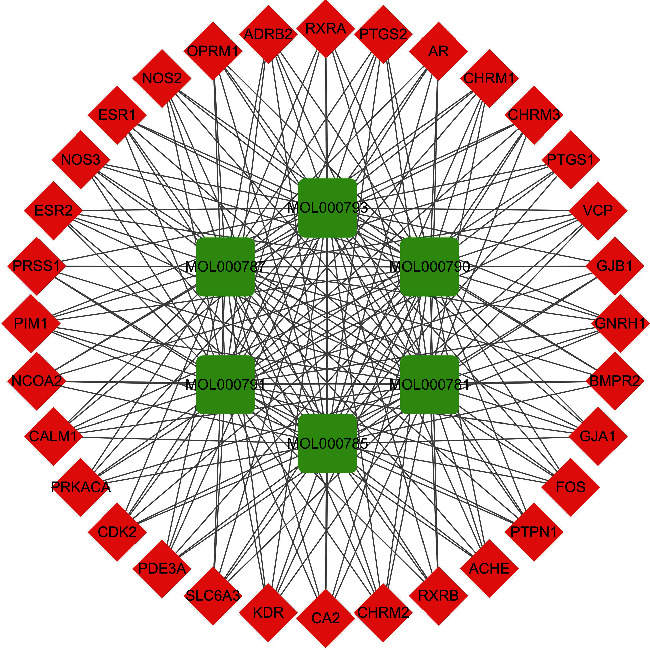
The ingredient-potential target network. The green rectangle represents the ingredients of *Corydalis decumbens* (Thunb.) Pers., and the red diamond represents the overlapping targets; the line between two nodes represents the interaction.

A total of 6 ingredients and 67 targets of *Corydalis decumbens* (Thunb.) Pers. were screened (Table 1, Figure 1, Table S1). A total of 3475 NSCLC target genes were obtained from the GeneCards database. After the comparative analysis, the overlapping genes, which might be potential therapeutic targets for NSCLC, were also obtained. The Venn diagram showed that 32 overlapping genes of *Corydalis decumbens* (Thunb.) Pers. participated in the regulation of NSCLC progression (Figure 2, Table S2). The generated visual PPI network of potential therapeutic targets for *Corydalis decumbens* (Thunb.) Pers. contained 38 nodes and 191 edges (Figure 3).

### 3.2. PPI Network Analysis

**Figure 4:**
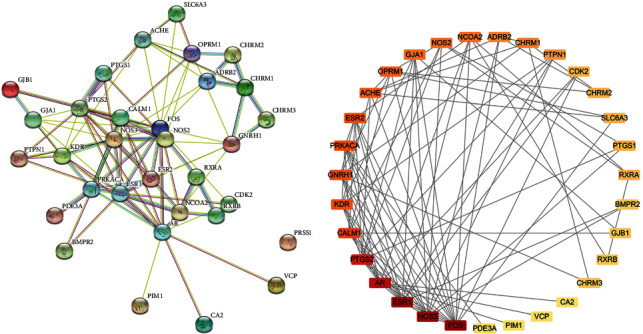
The protein-protein interaction network of overlapping genes. (a) Result from the STRING online tool. (b) Result from Cytoscape 3.7.1 software. The darker the color is, the greater the degree is.

Based on the potential pharmacodynamics of *Corydalis decumbens* (Thunb.) Pers. against NSCLC, the interacting proteins were screened using the STRING online tool. We found 31 nodes (PRSS1 did not interact with any of the genes) with 90 PPI relationships in the network (Figure 4(a)). The PPI network was constructed using Cytoscape 3.7.1 software for further visualization and analysis (Figure 4(b)). The results showed 15 genes whose degree was greater than or equal to 5.8 (average score) (Table S3) and 40 edges whose combined score was greater than or equal to 0.69 (average score) (Table S4). We found possible PPIs between *Corydalis decumbens* (Thunb.) Pers. and NSCLC-associated targets. The most likely association was between AR and NCOA2 (score = 0.999). The remaining interactions were as follows: NCOA2 (interacts with) RXRA, ESR1 (interacts with) NCOA2, CALM1 (interacts with) NOS2, CALM1 (interacts with) NOS3, ESR1 (interacts with) NOS3.

### 3.3. Functional Enrichment Analysis of *Corydalis decumbens* (Thunb.) Pers

**Figure 5:**
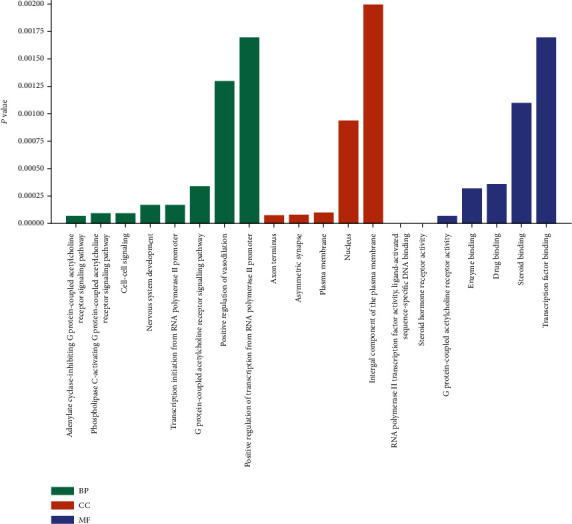
Gene Ontology analysis of potential target genes of *Corydalis decumbens* (Thunb.) Pers. against non-small cell lung cancer.

**Figure 6:**
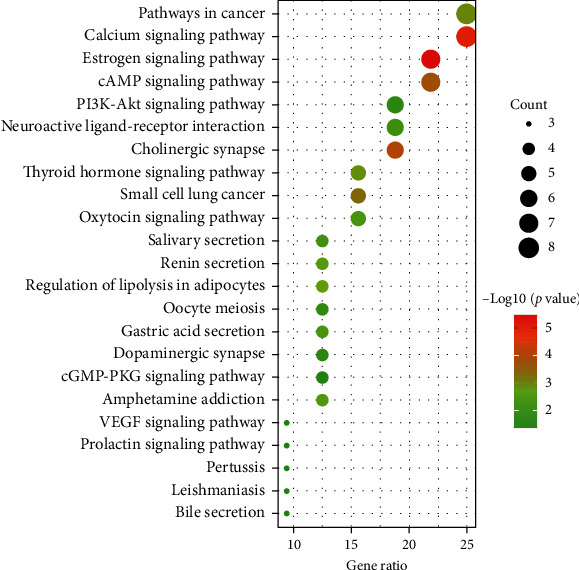
Pathway enrichment analysis of potential target genes of *Corydalis decumbens* (Thunb.) Pers. against non-small cell lung cancer using the Kyoto Encyclopedia of Genes and Genomics.

Potential targets of *Corydalis decumbens* (Thunb.) Pers. were used for further GO function and pathway enrichment analyses using the DAVID online tool. The results showed that the genes could be assigned to different GO terms, which mainly involved biological processes (BP) (90 items), cell components (CC) (27 items), and molecular functions (MF) (41 items). The cutoff for statistical significance was set *P* < 0.01, and the top 20 items with significant enrichment in biological processes, cell components, and molecular functions were screened, as shown in Figure 5. For biological processes, potential therapeutic targets of *Corydalis decumbens* (Thunb.) Pers. were significantly enriched in “adenylate cyclase-inhibiting G-protein coupled acetylcholine receptor signaling pathway” and “phospholipase C-activating G-protein coupled acetylcholine receptor signaling pathway.” For cell components, “axon terminus” and “asymmetric synapse” were mainly enriched. In addition, “RNA polymerase II transcription factor activity,” ligand-activated sequence-specific DNA binding,” “steroid hormone receptor activity”, and “G-protein coupled acetylcholine receptor activity” belonged to molecular functions. Additionally, the KEGG pathway enrichment analysis (*P* < 0.01) showed that these overlapping genes were mainly enriched in pathways such as the estrogen signaling pathway, calcium signaling pathway, cholinergic synapse, cAMP signaling pathway, PI3K-Akt signaling pathway, and VEGF signaling pathway (Figure 6).

### 3.4. Hub Genes Analysis

**Figure 7:**
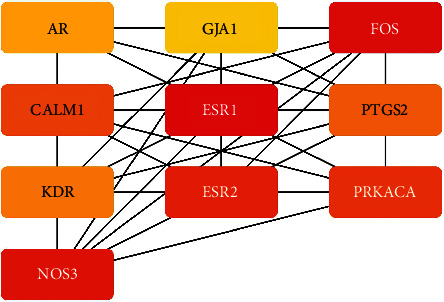
Top 10 hub genes of the potential target genes of *Corydalis decumbens* (Thunb.) Pers. against non-small cell lung cancer. The darker the color is, the greater the degree is.

**Figure 8:**
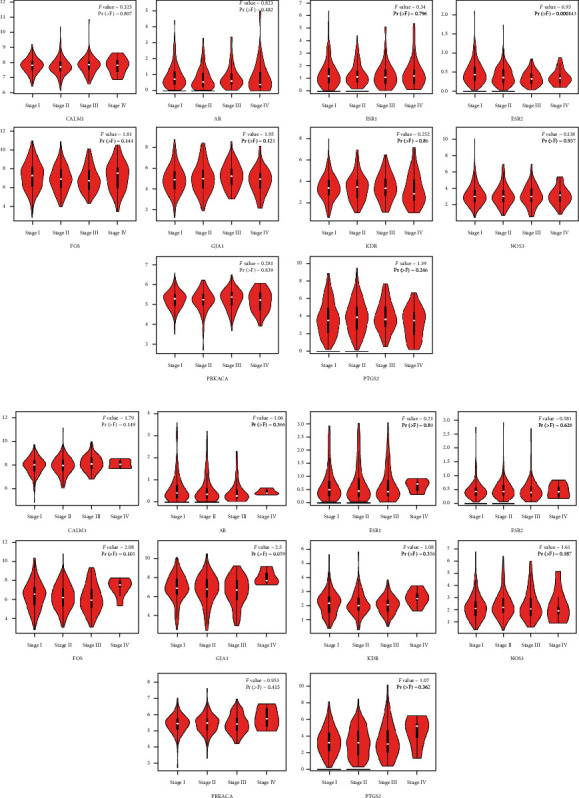
Correlation analysis among hub genes expression and tumor stage in non-small cell lung cancer patients. (a) LUAD patients. (b) LUSC patients.

**Figure 9:**
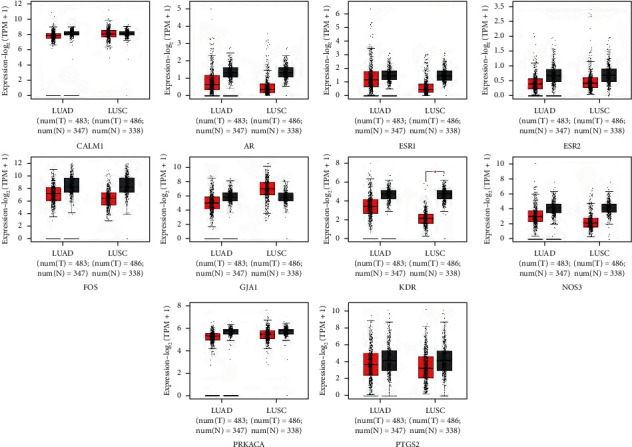
The expression of hub genes in non-small cell lung cancer. “*T*” indicates tumor tissue, and “*N*” indicates normal tissue.

**Figure 10:**
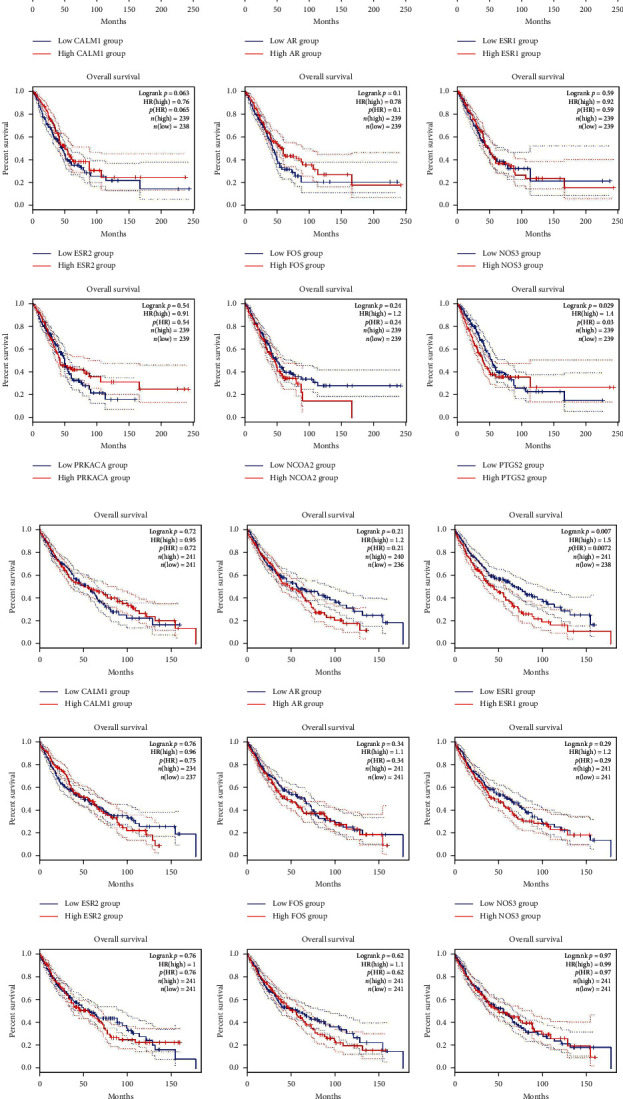
The prognostic value of mRNA of the hub factors in non-small cell lung cancer patients. (a) LUAD patients. (b) LUSC patients. The hub genes were *CALM1*, *AR*, *ESR1*, *ESR2*, *FOS*, *NOS3*, *PRKACA*, N*COA2*, and *PTGS2*.

The hub genes of *Corydalis decumbens* (Thunb.) Pers. for treating NSCLC were selected based on the Cytoscape 3.7.1 plugin cytoHubba (Figure 7). Potential therapeutic targets of *Corydalis decumbens* (Thunb.) Pers. included CALM1, AR, ESR1, ESR2, FOS, GJA1, KDR, NOS3, OPRM1, and PRKACA. Using the GEPIA dataset, we compared the mRNA expression of hub genes among NSCLC (LUAD and LUSC) at different stages. The results showed that not all of the *ESR2* mRNA expression levels were the same in different stage LUAD of patients (*P* < 0.05) (Figure 8). Additionally, we compared the transcriptional levels of hub genes between NSCLC and normal samples using the GEPIA dataset. The results indicated that the expression levels of *KDR* were lower in LUSC than in normal tissue (Figure 9). We further explored the critical efficiency of hub genes in the survival of patients with NSCLC. GEPIA “Survival analysis” tools were used to analyze the correlation of hub genes' mRNA levels in the survival of patients with NSCLC (LUAD and LUSC). The Kaplan–Meier curve and log-rank test analyses revealed that the expression of *PTGS2* was correlated with the median survival time of LUAD and the expression of *ESR1* was correlated with the median survival time of LUSC. The median survival time of the group with a high expression of *PTGS2* mRNA in LUAD was lower than that of the group with low expression of *PTGS2* (Figure 10(a)). The group with high expression of *ESR1* in LUSC had a lower median survival time than the group with low expression of *ESR1* (Figure 10(b)).

## 4. Discussion

Lung cancer is one of the most malignant cancers worldwide, and the effect of clinical treatment for this disease is not satisfactory [4]; therefore, this study explored the potential therapeutic effects of *Corydalis decumbens* (Thunb.) Pers. against NSCLC based on network pharmacology analysis and bioinformatics. Network pharmacology is an interdisciplinary subject that combines systems biology and pharmacology using high-throughput sequencing, genomics, and other technologies to analyze and explore the multichannel regulation of signaling pathways [14]. In this study, we predicted a potential PPI network between NSCLC and *Corydalis decumbens* (Thunb.) Pers. based on network pharmacology analysis. The results showed that the main ingredients of *Corydalis decumbens* (Thunb.) Pers. were PDSP1_000624, palmatine, fumarine, isocorypalmine, bicuculline, and C09367. Studies have shown that alkaloids exhibit good antitumor activity. Bicuculline can effectively inhibit the migration and invasion of human hepatocellular carcinoma MHCC97-H cells which is related to the downregulation of *VEGF*, *MMP-2*, and *MMP-9* gene expression and inhibition of JAK2/STAT3 signaling pathway activation [15]. Fumarine exerts antiproliferative effects by stimulating the p53 pathway in human colon cancer [16], and the apoptotic rate of HT-29 cells increased significantly after photodynamic treatment with palmatine [17].

A total of 32 overlapping genes were identified between the drug targets and NSCLC molecular targets. GO and KEGG analyses showed that they were mainly assigned to different GO terms and pathways. For example, transcription factor binding, protein binding, drug binding, estrogen signaling pathway, and calcium signaling pathway promote the occurrence and metastasis of NSCLC. Shi et al. [18] found that, in nonsmoking female patients with lung adenocarcinoma, Ca^2+^ is an important small-molecule signal that regulates cell function. The high expression of the Ca^2+^ pathway transcription factor NFATc2 is considered to be a new regulator for lung cancer to initiate cell phenotype, and high NFATc2 expression predicted poor tumor differentiation, adverse recurrence-free, and cancer-specific overall survivals in human lung cancers [19]. The increase in Ca^2+^ concentration in the cytoplasm of cancer cells can also induce apoptosis [20]. Increasing evidence suggests that the estrogen signaling pathway may be a therapeutic target for metastatic NSCLC [21]. Babita et al. [22] indicated that, in a preclinical model of NSCLC, estrogen promoted the growth of NSCLC and increased tumor microvessel density. In the estrogen signaling pathway, estrogen and estrogen receptors are thought to play an important role in NSCLC [23]. Hormone receptors are prognostic factors indicating metastatic tropism to the bones and comparably outcome in NSCLC early stage [24]. The PI3K-AKT signaling pathway plays a major role whole in the occurrence and development of NSCLC [25, 26]. Therefore, it is speculated that the potentially effective ingredients of *Corydalis decumbens* (Thunb.) Pers. mainly act on the key factors in the above pathways to effectively treat NSCLC.

Furthermore, we analyzed the top 10 potential therapeutic targets of *Corydalis decumbens* (Thunb.) Pers.: CALM1, AR, ESR1, ESR2, FOS, GJA1, KDR, NOS3, OPRM1, and PRKACA. The PPI network showed that the most likely association was between AR and NCOA2; NCOA2 and RXRA, ESR1 and NCOA2, FOS and ESR2, ESR2 and NCOA2, and FOS and ESR2. In tumor models, the adrenaline signaling pathway plays a regulatory role in tumor growth and development. Moreover, the activation of *β*-adrenergic receptors promotes tumor progression and development of resistance to treatment [27, 28]. Fos/Jun-dependent signal transduction pathways are thought to be major effects of oncogene action in NSCLC [29]; moreover, exosome-derived miR-224-5p induced NSCLC cell proliferation and metastasis by directly suppressing *AR* [30]. Studies have found that vascular epidermal growth factor receptor 2 (VEGFR-2) plays a key role in the occurrence and development of tumors including NSCLC [31–33]. VEGFR-2 is also known as the KDR in the human body. Several studies have shown that the estrogen receptor beta (ESR2) appeared in many tumors, including lung cancer, wherein ESR2 and interleukin 6 receptor (IL-6R) interacts to mediate lung cancer progression. Cell proliferation, invasion, and cell cycle were significantly increased, and cell apoptosis was markedly inhibited by the concurrent action of ESR2 and IL-6 in A549 cells [34]. Chen et al. [35] showed that phosphorylated ESR1 could directly bind to the promoter of FOSL1 (FOS-like 1) in ESCs/DSCs. Moreover, silencing of ESR1 expression indirectly abrogated FOSL1 expression at the transcript and protein levels.

## 5. Conclusions

This study is used the network pharmacology analysis to understand the mechanism of action of *Corydalis decumbens* (Thunb.) Pers. and preliminarily verified the effects of *Corydalis decumbens* (Thunb.) Pers. in multicomponent, multitarget, and multichannel treatment of lung cancer. The application of network pharmacology revealed the potential mechanism underlying the treatment of NSCLC by *Corydalis decumbens* (Thunb.) Pers. and provides a theoretical basis for further in-depth research. However, this study did not consider the influence of drug dosage, drug ingredients, and drug delivery methods on the treatment results. Therefore, further experimental study is required to verify the results of this study.

## Figures and Tables

**Table 1:** Information of *Corydalis decumbens* (Thunb.) Pers. ingredients.

Mol. ID	Name	OB (100%)	DL
MOL000781	PDSP1_000624	33.45	0.69
MOL000785	Palmatine	64.6	0.65
MOL000787	Fumarine	59.26	0.83
MOL000790	Isocorypalmine	35.77	0.59
MOL000791	Bicuculline	69.67	0.88
MOL000793	C09367	47.54	0.69

## Data Availability

The data used to support the findings of this study are included within the Supplementary Materials.

## Supplementary Materials

Supplementary Materials Table S1 is about the information of ingredients corresponding target. Table S2 is a description of overlapping genes. Table S3 is the overlapping genes degree score information. Table S4 described the combined score of interaction genes.
