# Supplementary material for: Network Pharmacology-Based Analysis of the Effects of Corydalis decumbens (Thunb.) Pers. in Non-Small Cell Lung Cancer
Source: Evid Based Complement Alternat Med. 2021 Aug 21;2021:4341517. doi: 10.1155/2021/4341517 (PMC8421182; doi:10.1155/2021/4341517)
Supplement: Supplementary Materials — Table S1 is about the information of ingredients corresponding target. Table S2 is a description of overlapping genes. Table S3 is the overlapping genes degree score information. Table S4 described the combined score of interaction genes. [file 4341517.f1.docx]

**Table S1 The information of ingredients** [**corresponding**](http://www.youdao.com/w/corresponding/#keyfrom=E2Ctranslation) **target**

| No. | Target name | Gene name |
| --- | --- | --- |
| 1 | Prostaglandin G/H synthase 1 | PTGS1 |
| 2 | Muscarinic acetylcholine receptor M3 | CHRM3 |
| 3 | Muscarinic acetylcholine receptor M1 | CHRM1 |
| 4 | Androgen receptor | AR |
| 5 | Sodium channel protein type 5 subunit alpha | SCN5A |
| 6 | Muscarinic acetylcholine receptor M5 | CHRM5 |
| 7 | Prostaglandin G/H synthase 2 | PTGS2 |
| 8 | Retinoic acid receptor RXR-alpha | RXRA |
| 9 | Delta-type opioid receptor | OPRD1 |
| 10 | Alpha-1B adrenergic receptor | ADRA1B |
| 11 | Beta-2 adrenergic receptor | ADRB2 |
| 12 | Alpha-1D adrenergic receptor | ADRA1D |
| 13 | DNA topoisomerase II | TOP 2A |
| 14 | Mu-type opioid receptor | OPRM1 |
| 15 | Nitric oxide synthase, inducible | NOS2 |
| 16 | Potassium voltage-gated channel subfamily H member 2 | KCNH2 |
| 17 | Estrogen receptor | ESR1 |
| 18 | Nitric-oxide synthase, endothelial | NOS3 |
| 19 | Estrogen receptor beta | ESR2 |
| 20 | Heat shock protein HSP 90 | HSP90 |
| 21 | Trypsin-1 | PRSS1 |
| 22 | Proto-oncogene serine/threonine-protein kinase Pim-1 | PIM1 |
| 23 | Nuclear receptor coactivator 2 | NCOA2 |
| 24 | Calmodulin | CALM1 |
| 25 | mRNA of PKA Catalytic Subunit C-alpha | PRKACA |
| 26 | Cell division protein kinase 2 | CDK2 |
| 27 | Coagulation factor VII | F7 |
| 28 | Coagulation factor Xa | F10 |
| 29 | 5-hydroxytryptamine receptor 3A | HTR3A |
| 30 | Muscarinic acetylcholine receptor M4 | CHRM4 |
| 31 | 5-hydroxytryptamine 2A receptor | HTR2A |
| 32 | Sodium-dependent serotonin transporter | SLC6A4 |
| 33 | Voltage-dependent L-type calcium channel subunit alpha-1S | CACNA1S |
| 34 | CGMP-inhibited 3',5'-cyclic phosphodiesterase A | PDE3A |
| 35 | Sodium-dependent dopamine transporter | SLC6A3 |
| 36 | Type IV phosphodiesterase | PDE1C |
| 37 | Dopamine D1 receptor | DRD1 |
| 38 | Vascular endothelial growth factor receptor 2 | KDR |
| 39 | D(1B) dopamine receptor | DRD5 |
| 40 | Carbonic anhydrase II | CA2 |
| 41 | Alpha-2C adrenergic receptor | ADRA2C |
| 42 | Sodium-dependent noradrenaline transporter | SLC6A2 |
| 43 | Alpha-1A adrenergic receptor | ADRA1A |
| 44 | 5-hydroxytryptamine 2C receptor | HTR2C |
| 45 | Muscarinic acetylcholine receptor M2 | CHRM2 |
| 46 | Alpha-2B adrenergic receptor | ADRA2B |
| 47 | D(3) dopamine receptor | DRD3 |
| 48 | Retinoic acid receptor RXR-beta | RXRB |
| 49 | Ig gamma-1 chain C region | Ighg1 |
| 50 | Nuclear receptor coactivator 1 | NCOA1 |
| 51 | Calcium-activated potassium channel subunit alpha 1 | KCNMA1 |
| 52 | Thrombin | F2 |
| 53 | Acetylcholinesterase | ACHE |
| 54 | mRNA of Protein-tyrosine phosphatase, non-receptor type 1 | PTPN1 |
| 55 | Proto-oncogene c-Fos | FOS |
| 56 | Gap junction alpha-1 protein | GJA1 |
| 57 | Gamma-aminobutyric acid type B receptor subunit 1 | GABBR1 |
| 58 | Bone morphogenetic protein receptor type-2 | BMPR2 |
| 59 | Metabotropic glutamate receptor 5 | GRM5 |
| 60 | Progonadoliberin-1 | GNRH1 |
| 61 | Aldehyde dehydrogenase, dimeric NADP-preferring | ALDH3A1 |
| 62 | Gonadotropin-releasing hormone receptor | GNRHR |
| 63 | Prostaglandin G/H synthase 2 | CRH |
| 64 | Muscarinic acetylcholine receptor M1 | NMDE4 |
| 65 | Muscarinic acetylcholine receptor M3 | GJB1 |
| 66 | Androgen receptor | GRM1 |
| 67 | Transitional endoplasmic reticulum ATPase | VCP |

**Table S2 The overlapping genes information**

| **No.** | **1** | **2** | **3** | **4** | **5** | **6** | **7** | **8** | **9** | **10** | **11** | **12** | **13** | **14** | **15** | **16** |
| --- | --- | --- | --- | --- | --- | --- | --- | --- | --- | --- | --- | --- | --- | --- | --- | --- |
| **Gene Name** | PTGS1 | CHRM3 | CHRM1 | AR | PTGS2 | RXRA | ADRB2 | OPRM1 | NOS2 | ESR1 | NOS3 | ESR2 | PRSS1 | PIM1 | NCOA2 | CALM1 |
| **No.** | **17** | **18** | **19** | **20** | **21** | **22** | **23** | **24** | **25** | **26** | **27** | **28** | **29** | **30** | **31** | **32** |
| **Gene Name** | PRKACA | CDK2 | PDE3A | SLC6A3 | KDR | CA2 | CHRM2 | RXRB | ACHE | PTPN1 | FOS | GJA1 | BMPR2 | GNRH1 | GJB1 | VCP |

**Table S3 The overlapping genes degree score**

| Gene name | FOS | NOS3 | ESR1 | AR | PTGS2 | CALM1 | KDR | GNRH1 | PRKACA | ESR2 | ACHE | OPRM1 | NCOA2 | NOS2 | GJA1 |
| --- | --- | --- | --- | --- | --- | --- | --- | --- | --- | --- | --- | --- | --- | --- | --- |
| Degree | 15 | 14 | 14 | 12 | 11 | 9 | 8 | 8 | 8 | 8 | 7 | 7 | 6 | 6 | 6 |

|  |  |  |  |  |  |  |  |  |  |
| --- | --- | --- | --- | --- | --- | --- | --- | --- | --- |

**Table S4 The combined score of interaction genes**

| Interaction gene | Combined_score |
| --- | --- |
| AR (interacts with) NCOA2 | 0.999 |
| NCOA2 (interacts with) RXRA | 0.997 |
| ESR1 (interacts with) NCOA2 | 0.995 |
| CALM1 (interacts with) NOS2 | 0.992 |
| CALM1 (interacts with) NOS3 | 0.985 |
| ESR1 (interacts with) NOS3 | 0.985 |
| ESR1 (interacts with) FOS | 0.982 |
| KDR (interacts with) NOS3 | 0.982 |
| ESR2 (interacts with) NCOA2 | 0.971 |
| ESR2 (interacts with) NOS3 | 0.967 |
| ESR2 (interacts with) FOS | 0.953 |
| ESR1 (interacts with) ESR2 | 0.941 |
| NCOA2 (interacts with) RXRB | 0.933 |
| ADRB2 (interacts with) CHRM2 | 0.919 |
| CHRM2 (interacts with) OPRM1 | 0.918 |
| ADRB2 (interacts with) CHRM1 | 0.913 |
| CHRM1 (interacts with) GNRH1 | 0.913 |
| CHRM1 (interacts with) CHRM3 | 0.911 |
| NOS2 (interacts with) RXRA | 0.909 |
| AR (interacts with) PRKACA | 0.906 |
| NOS2 (interacts with) PTGS2 | 0.906 |
| CHRM3 (interacts with) GNRH1 | 0.905 |
| RXRA (interacts with) RXRB | 0.905 |
| KDR (interacts with) PRKACA | 0.901 |
| NOS3 (interacts with) PRKACA | 0.900 |
| FOS (interacts with) PRKACA | 0.892 |
| PTGS1 (interacts with) PTGS2 | 0.889 |
| ESR1 (interacts with) PRKACA | 0.871 |
| AR (interacts with) GNRH1 | 0.849 |
| NOS3 (interacts with) PTGS2 | 0.835 |
| FOS (interacts with) PTGS2 | 0.817 |
| CHRM1 (interacts with) CHRM2 | 0.813 |
| NOS2 (interacts with) NOS3 | 0.813 |
| FOS (interacts with) GNRH1 | 0.811 |
| PDE3A (interacts with) PRKACA | 0.810 |
| ESR2 (interacts with) PRKACA | 0.800 |
| FOS (interacts with) OPRM1 | 0.792 |
| ESR1 (interacts with) PTGS2 | 0.773 |
| ESR1 (interacts with) GNRH1 | 0.757 |
| CDK2 (interacts with) ESR1 | 0.718 |
| CALM1 (interacts with) PRKACA | 0.690 |
